# Transcriptomic responses of corpuscle of Stannius gland of Japanese eels (*Anguilla japonica*) to Changes in Water Salinity

**DOI:** 10.1038/srep09836

**Published:** 2015-04-24

**Authors:** Jie Gu, Jing-Woei Li, William Ka-Fai Tse, Ting-Fung Chan, Keng-Po Lai, Chris Kong-Chu Wong

**Affiliations:** 1 Department of Biology, Hong Kong Baptist University, Hong Kong SAR; 2 School of Life Sciences, Hong Kong Bioinformatics Centre, The Chinese University of Hong Kong, Hong Kong SAR; 3 School of Biological Sciences, Kadoorie Biological Sciences Building, The University of Hong Kong, Pokfulam Road, Hong Kong SAR

## Abstract

Physiological studies of a unique endocrine gland in fish, named corpuscles of Stannius (CS), described a Ca2^+^-regulatory function for this gland mediated by stanniocalcin-1, a hypocalcemic polypeptide hormone. However, to date, the endocrine functions of the glands have not been completely elucidated.

We hypothesized that other unidentified active principles in the glands are involved in the regulation of plasma ion (Na^+^, Ca2^+^) and/or blood pressure. In this study, transcriptome sequencing of CS glands was performed using Japanese eels (*Anguilla japonica*) adapted to freshwater (FW) or seawater (SW) to reveal the presence and differential expression of genes encoding proteins related to the ion-osmoregulatory and pressor functions. We acquired a total of 14.1 Mb and 12.1 Mb quality-trimmed reads from the CS glands collected from FW and SW adapted eels, respectively. The *de novo* assembly resulted in 9254 annotated genes. Among them, 475 genes were differentially expressed with 357 up- and 118 down-regulated in the SW group. Gene ontology analysis further demonstrated the presence of natriuresis and pressor related genes. In summary, ours is the first study using high-throughput sequencing to identify gene targets that could explain the physiological importance of the CS glands.

In 1839, the German zoologist H. Stannius identified an endocrine gland on the ventral surface of fish kidneys^1^. Stannius thought that the glands were equivalent to the mammalian adrenal glands due to their anatomical position. The name of the glands, corpuscle of Stannius (CS), was coined in 1847^2^ and the assumption that this gland was an adrenal gland was maintained until the 20^th^ century. In 1942, the ontogeny of the CS glands was reported. It was discovered that the glands were histologically distinct from the piscine interrenal and chromaffin tissues^3^. No steroidogenic activity was detected in these glands^4^. Electron microscopic studies revealed that the CS cells possessed cytoplasmic features of polypeptide hormone-secreting cells^5^. Accordingly, CS was confirmed to be a unique endocrine gland found only in fish.

Surgical removal of the CS glands (stannioectomized, STX) from fishes causes plasma hypercalcemia^6^ as well as a reduction of plasma Na^+^ and Cl^-^ levels^7^,^8^, and a decrease in dorsal aortic blood pressure^9^. Intriguingly, STX fishes that were maintained in low-calcium water did not suffer from the rise of serum Ca^2+^ levels. These observations indicated that the ambient water was the major source of the Ca^2+^ responsible for hypercalcemia in the STX fishes. Consequently, fish gills were suggested to be the main tissue for Ca^2+^ absorption from the ambient water. This hypothesis was later supported by a study conducted by Fenwick and So^10^ who demonstrated that the rate of gill calcium transport (GCAT) was significantly increased in STX fishes. Conversely, the increase of GCAT can be reduced by injection of CS extracts^10^,^11^,^12^. This finding indicated that the active principle(s) from the CS extracts contained “inhibitory factor(s)” that can reduce the rate of GCAT^10^,^13^. One of the major active principles in CS gland extracts, a hypocalcemic hormone named stanniocalcin-1 (STC-1), was identified in 1980s^14^,^15^,^16^,^17^. However, the characteristic and significance of other substance(s) with ion-osmoregulatory and pressor functions remain unknown^18^,^19^,^20^,^21^.

Previous physiological studies had demonstrated that CS glands are involved in the regulation of blood pressure and natriuresis^22^; however, there is limited information regarding this regulatory role of CS glands, to date. Thus, we hypothesized that there might be some unidentified active principles in the CS glands associated with these reported physiological functions. In this study, a high-throughput transcriptome sequencing (RNA-seq) approach was adopted to investigate the transcriptome profiles of the CS glands from fish adapted to freshwater or seawater environments. The differential expression patterns of the CS glands were compared and the genes involved in Ca^2+^ metabolism, ion-osmoregulation, and blood pressure were identified. This study provides an important resource for future investigations on CS glands functions.

## Methods

### Maintenance of Japanese eels (*Anguilla japonica*)

The methods were carried out in Hong Kong Baptist University in accordance with the approved guidelines. All experimental procedures were approved by the Hong Kong Baptist University, Hong Kong Special Administrative Region. Japanese eels (*A. japonica*) weighing between 500–600g, were reared in fiberglass tanks supplied with charcoal-filtering aerated tap-water (freshwater, FW) at 18–20°C under a 12 h: 12 h L:D photoperiod for at least 2 weeks of acclimation before the experiments. The fish were then either maintained in FW (n = 5) or transferred to seawater (SW) (n = 5) for another two weeks. After this, the fish were anesthetized with 0.1% MS-222 (Sigma) for the collection of the CS glands.

### RNA Isolation, cDNA Library Construction, and Illumina Deep Sequencing

Total RNA was isolated from the CS glands of fish using TRIzol reagent (Life Technologies, CA, USA). The RNA concentration was measured using Qubit® RNA Assay Kit in Qubit® 2.0 Fluorometer (Life Technologies, CA, USA). RNA samples (300 ng) with a RNA Integrity Number (RIN) greater than 8, as determined by the Agilent 2100 Bioanalyzer system (Agilent Technologies, CA, USA), were used for library construction. Four independent libraries were prepared for RNA sequencing. Briefly, the cDNA libraries were prepared using the TruSeq Stranded mRNA LT Sample Prep Kit (Illumina, San Diego, USA) following the manufacturer’s protocol. Index codes were ligated to identify individual samples. mRNA was purified from the total RNA using poly-T oligo-attached magnetic beads (Illumina, San Diego, USA), and, then, fragmented using divalent cations under elevated temperature in the Illumina fragmentation buffer. First and second strand cDNAs were synthesized using random oligonucleotides and SuperScript II, followed by DNA polymerase I and RNase H. Overhangs were blunted by using exonuclease/polymerase and, after 3**’** end adenylation, Illumina PE adapter oligonucleotides were ligated. DNA fragments that ligated with adaptor molecules on both ends were enriched using the Illumina PCR Primer Cocktail in a 15-cycle PCR reaction. Products were purified and quantified using the AMPure XP and the Agilent Bioanalyzer 2100 systems, respectively. Before sequencing, the libraries were normalized and pooled together in a single lane on an Illumina MiSeq platform. Paired-end reads, each of 150-bp read-length, were sequenced. Adapters and reads containing poly-N were first trimmed and the sequence-reads were dynamically trimmed according to BWA's − q algorithm^23^*.* Briefly*,* a running sum algorithm was executed in which a cumulative area-plot is plotted from 3’-end to the 5’-end of the sequence reads and where positions with a base-calling Phred quality lower than 30 cause an increase of the area and vice versa. Such plot was built for each read individually and each read was trimmed from the 3**’**-end to the position where the area was greatest*.* Read-pairs were then synchronized such that all read-pairs with sequence on both sides longer than 35 bp after quality trimming were retained. Any singleton read resulting from read trimming was removed^23^. All the downstream analyses were based on quality-trimmed reads.

### De novo Transcriptome assembly

Forward and reverse reads from all the libraries/samples were pooled and subjected to transcriptome *de novo* assembly using Trinity (version r2013-02-25) with “min_kmer_cov” set to 2 and all other parameters set to default^24^. Trinity uses fixed *k*-mer to generate an assembly and it is efficient in recovering full-length transcripts as well as spliced isoforms.

### Annotation of assembled transcripts

Coding sequences (open reading frames, ORF) were identified by Transdecoder^25^ using the following criteria: (1) the longest ORF was identified within each transcript; (2) from the longest ORFs extracted, a subset of the longest ones was identified and randomized to provide a sequence composition corresponding to non-coding sequences before being used to parameterize a Markov model based on hexamers; and (3) all the longest ORFS were scored according to the Markov Model to identify the highest scoring reading-frame out of the six possible reading-frames. These ORF were then translated to protein sequences and subjected to (1) BLASTp search against UniProtKB/Swiss-Prot with a cut-off e-value of 1.0 × 10^-6^^26^,^27^, (2) protein domain search via HMMScan, (3) transmembrane helicase prediction by TMHMM, and (4) signal peptide prediction by SignalP.

### Comparative analysis of *Anguilla japonica* transcripts and genome annotation of transcripts

Annotated proteins of *Anguilla* species (Taxon identifier: 7935) with GO term “hormone activity” (GO: 0005179) were retrieved from the UniProt database on October 31, 2014. mRNA transcripts of *Anguilla* species were retrieved from NCBI ("Anguilla"[Organism] AND biomol_mrna[PROP]). Draft genome sequences of *Anguilla japonica (A. japonica*) and *Anguilla anguilla (A. anguilla),* and predicted cDNAs of *A. anguilla* were retrieved from the ZF-Genomics database (http://www.eelgenome.com/)^28^. A transcriptome assembly of *A. anguilla* as well as an Eeelbase-specific microarray targeting *A. anguilla* transcripts were retrieved from the Eeelbase database (http://compgen.bio.unipd.it/eeelbase/)^29^,^30^.

Transcripts from *A. japonica* and *A.* *anguilla* were considered orthologous if they were the symmetrical best hits in each reciprocal all-against-all BLASTn search (i.e. Reciprocal Best Hit)^31^. Briefly, orthologs to the *A. anguilla* sequences were identified first by comparing the assembled transcript to the database using BLAST search. The highest-scoring hit was obtained and, then, a BLAST search was run against the database of the assembled transcripts. The hit in *A. anguilla* sequences was considered an ortholog of the assembled transcript if and only if the second BLAST search returned the assembled transcript that was the highest scorer in the first BLAST search. The transcriptome assembly was aligned to the draft genome of *A. japonica* and *A.* *anguilla* using GMAP (version 2014-08-04) with the parameter –no-chimeras and –cross-species^32^.

### Differential expression and GO enrichment analysis

In our analysis, differential gene expression and TMM-normalized FPKM gene expression were calculated separately. This is because RSEM does not support gapped alignment, and the alignment accuracy of Bowtie used by RSEM is known to be lower than that of other aligners^33^, thereby hindering the use of the alignments produced by other aligners. Sequencing reads were mapped to the assembled transcripts using Novoalign (v3.00.05) with parameter –r ALL to report all multi-mapped reads (http://www.novocraft.com/). Alignment files were sorted using Samtools (http://samtools.sourceforge.net/) to generate a read-name sorted BAM file. Then, “Samtools view -F 0x4” was used to parse the mapped reads from the BAM file and the number of read-pairs mapping to each transcript in each sample were summarized to generate a count table (http://seqanswers.com/forums/showthread.php?t=29745)^34^. Ambiguously mapped read-pairs with each end mapped to different transcripts were discarded. Read-count data were then subjected to differential expression analysis using the edgeR package^35^. Samples with identical treatments were considered to be biological replicates. Genes with B&H corrected *p*-value <0.05 and log2 (fold change) >1 were consider to show statistically significant differential expression. RSEM pipeline was used to independently calculate TMM-normalized-FPKM expression values^36^. Following this calculation, we cross-checked our edgeR results with those generated by the RSEM pipeline. Dysregulated genes were subjected to KEGG pathway analysis using DAVID Tools to decipher the molecular interaction networks that might be deregulated^37^.

### Quantitative real-time PCR (qRT-PCR)

To validate the sequencing data, an independent cohort of FW or SW (n = 3) adapted fish were sampled. The differentially expressed genes were selected for qRT-PCR analysis. Briefly, total cellular RNA (0.5μg) was reversed transcribed using the high capacity RNA-to-cDNA kit (Applied Biosystems, Foster City, CA, USA). qRT-PCR reactions were conducted using the Power SYBR^®^ green PCR master mix with the StepOne^™^ real-time PCR system (Life Technologies, CA, USA). Verified gene-specific primers (Table S1) of *A. japonica* were used. The occurrence of primer-dimers and secondary products was inspected using melting curve analysis. Our data indicated that the amplification was specific for each individual set of primers and control amplification was done either without reverse transcriptase or without RNA. *gadph* was used as a housekeeping gene and the relative expression ratio of *target gene/gapdh* was calculated according to the method described by Pfaffl^38^:

Expression ratio = E*_target_*^CP*target* (control–treatment) ^/ E*_gapdh_*^CP*gapdh* (control–treatment)^, where E = 10^(–1/slope)^ and CP is the crossing point at which fluorescence rises above the background level.

### Availability of supporting data

The sequencing data from this study have been submitted to the NCBI Sequence Read Archive (SRA) (http://www.ncbi.nlm.nih.gov/sra) under the accession number **SRP049701**.

## Results

### Workflow of the study

**Figure 1:**
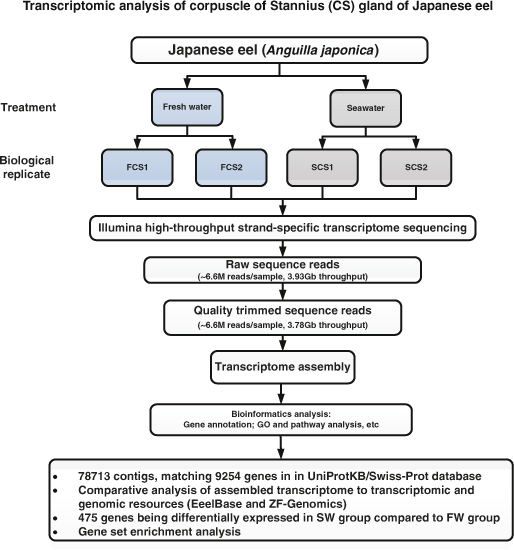
Workflow of Illumina deep sequencing and bioinformatic analyses. It includes sample preparation, cDNA library construction, Illumina sequencing, and data analyses including transcriptome assembly, BLAST search, GO annotation, and gene expression analysis.

In this study, a pair of CS glands from each fish was used to prepare one pooled RNA sample. Two biological replicates of FW and SW adapted fish were performed. Four cDNA libraries were constructed and subjected to Illumina transcriptome sequencing. **The overall workflow of the study is shown in (**Fig. 1)**.**

### Illumina RNA-Seq and de novo transcriptome Assembly

**Figure 2:**
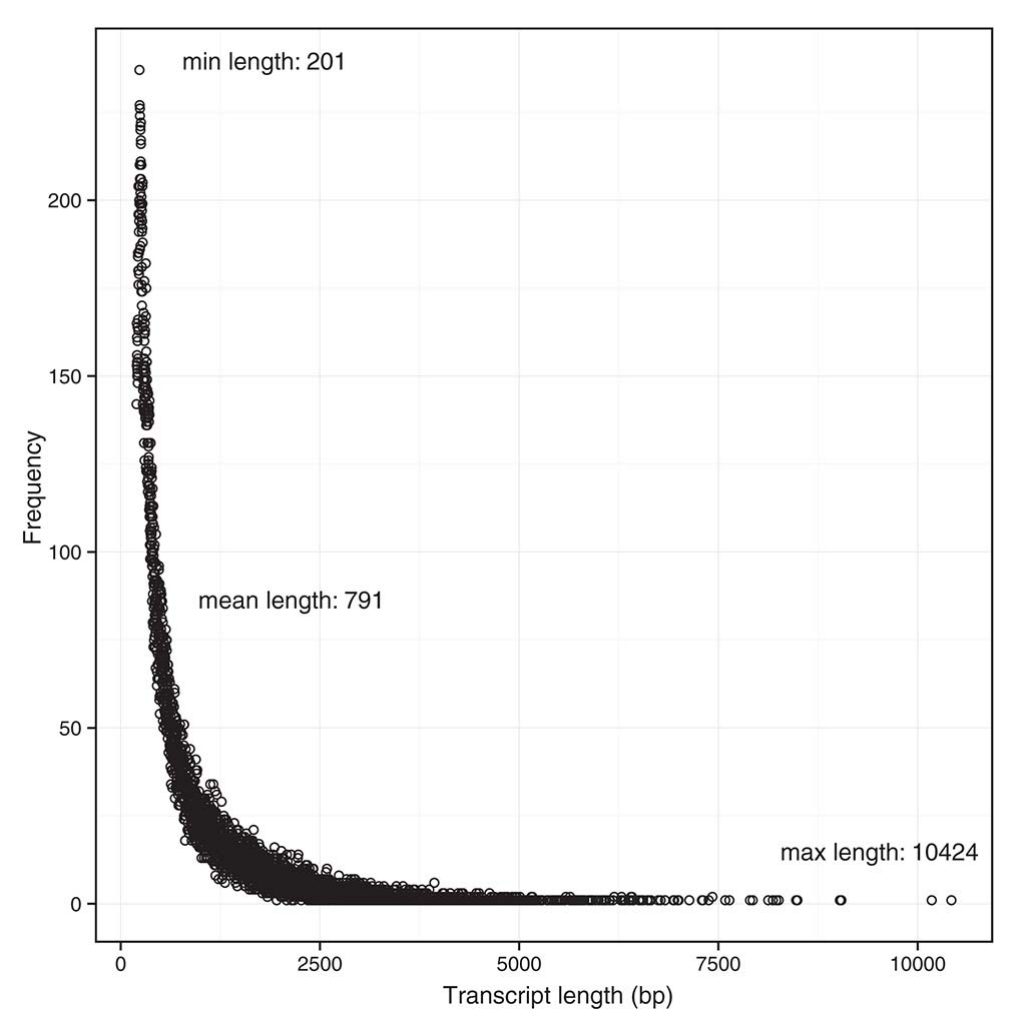
Distribution of the assembled transcript length. The transcripts length ranges from 201 bp to 10424 bp. The average length was 791 bp. X-axis indicates the transcript length (nt), and the Y-axis indicates the number of assembled transcripts of the particular length indicated by the X-axis.

We obtained 7.22 Mb and 6.92 Mb quality-trimmed Illumina reads from the FW CS gland samples (FCS1 and FCS2, respectively) and, 6.61 Mb and 5.47 Mb quality-trimmed Illumina reads from the SW CS gland samples (SCS1 and SCS2, respectively). A total of 2.05 Gb and 1.73 Gb of clean bases were obtained from the FW and SW samples, respectively. The *de novo* transcriptome was formed by 78713 contigs with an average contig length of 791 bp (the shortest sequence was 201 bp and the longest one was 10424 bp) (Fig. 2).

### Gene annotation

**Figure 3:**
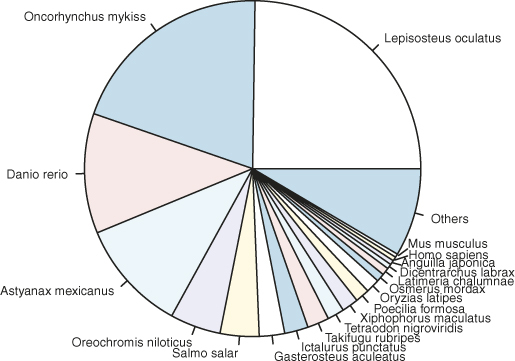
Species distribution of matched genes. The unigenes compared to the UniProtKB database, 31.35% of the matched genes showed similarities with *Lepisosteus oculatus*, followed by *Oncorhynchus mykiss* (19.92%), *Danio rerio* (11.55%), *Astyanax mexicanus* (10.77%), *Oreochromis niloticus* (4.88%), *Salmo salar* (3.74%), *Gasterosteus aculeatus* (2.44%), *Ictalurus punctatus* (2.27%), *Takifugu rubripes* (2.10%), and others (15.95%).

The assembled transcripts were subjected to 6-frame translations and the data of likely coding sequences were extracted. These likely coding sequences were randomized to provide a sequence composition corresponding to non-coding sequences. All the longest ORFs were scored according to the Markov Model (log likelihood ratio based on coding/noncoding) in each of the six possible reading frames. If the putative ORF proper coding frame scored positive and was the highest among the other presumed wrong reading frames, then that ORF was reported. If a high-scoring ORF was eclipsed by a longer ORF in a different reading frame, it would be excluded. Annotation analysis were implemented to compare the predicted ORF sequences against the UniProtKB/Swiss-Prot database using BLASTp search with a cut-off e-value of 1.0 × 10^-6^. In this study, 9254 genes were matched to the UniProtKB/Swiss-Prot database (Table S2). Regarding the taxonomic distribution of the genes, according to the UniProtKB/TrEMBL database, 24.78% of the matched genes showed similarities with *Lepisosteus oculatus*, followed by *Oncorhynchus mykiss* (19.92%), *Danio rerio* (11.55%), *Astyanax mexicanus* (10.77%), *Oreochromis niloticus* (4.88%), *Salmo salar* (3.74%), *Gasterosteus aculeatus* (2.44%), *Ictalurus punctatus* (2.27%), *Takifugu rubripes* (2.10%), and others (15.95%) (Fig. 3). In fact, as of the date when the analysis was performed, only 1238 and 80 protein sequences of *Anguilla* species were deposited in the UniProtKB/TrEMBL and UniProtKB/Swiss-Prot databases, respectively. Therefore, it was not surprising to observe so few hits to *Anguilla* species, which was probably due to the under-representation of *Anguilla* species protein sequences in the UniProt database.

### Comparative analysis of *A. japonica* transcripts with various eel species

We first compared our unified transcriptome assembly to the existing transcriptome resources of eel species. Eel endocrinology has been a subject of interest for a long time and, thus, numerous eel hormone sequences are available. To investigate what kind of hormones were expressed in the CS glands, we compared our assembled transcriptome to the existing eel protein sequences with hormone activity and found calcitonin, stanniocalcin, activin, adrenomedullin, insulin-like growth factor, natriuretic peptide, relaxin, urotensin, and ventricular natriuretic peptide in the CS gland transcriptome (Table S3). We then sought to study the expression of annotated eel genes in our CS gland-specific transcriptome. In order to accomplish this goal, we performed both nucleotide and protein level searches in our assembled transcriptome. The nucleotide search suggested that 39.2% of known transcripts discovered in various eel species are present in the CS gland of *A. japonica* (Table S4). We found that 27.8% of the proteins annotated in eel species by UniProt/TrEMBL non-reviewed database are present in our transcriptome assembly (Table S5).

The EeelBase database provides transcriptome resources of *A. anguilla* generated from 640,040 reads sequenced by both 454 and Sanger technologies^29^,^30^. We compared our *A. japonica* transcriptome assembly to that of the Eeelbase database using BLASTn search, with e-value <1E-5, identity ≥0.95, and number of aligned nucleotides ≥50%. It was found that 4085 *A. anguilla* transcripts annotated in the EeelBase database are present in our *A. japonica* assembly. The Eeelbase database has also developed an Eeelbase specific microarray^29^ that targets a subset (~33%) of transcripts among their assembled *A.* *anguilla* transcriptome. The Eeelbase specific microarray targets *A. anguilla* transcripts that matched 2293 transcripts of our *A. japonica* assembly. Since the specificity of the microarray probe depends on the hybridization of the probe sequences to the cDNA to be probed, we required the alignment length threshold to be at least 90%, with an identity of at least 95%. Using these parameters among the 2293 transcripts, we estimated that only 25% (~582) *A. japonica* transcripts could be probed uniquely by the Eeelbase specific microarray (Table S6).

Orthologous transcripts between *A. japonica* and *A.* *anguilla* were identified by comparing our assembled transcriptome to the predicted cDNA of the eel species. We decided against the use of only cDNAs from *A. anguilla* because of the low-coverage sequencing depth of the available *A. anguilla* transcriptome in the EeelBase database. Based on reciprocal BLAST searches, 19382 putative homologs between *A. japonica* and *A.* *anguilla* were identified (Table S7).

With the availability of the draft genomes of *A. japonica* and *A.* *anguilla*, we sought to determine the gene structure of our assembled *A. japonica* transcriptome by aligning the assembled transcripts to the draft genomes. Regarding the *A. japonica* genome*,* we found that the majority (89%; 69920/78713) of the transcripts could be aligned. In fact, 82% (64601/78713) of the transcripts were almost completely aligned (≥95% of the length of assembled transcripts). Regarding the *A. anguilla* genome*,* we found that the majority (78%; 61737/78713) of the transcripts could also be aligned. In fact, 70% (55078/78713) of the transcripts were almost completely aligned (≥95% of the length of assembled transcripts). Based on both *A. japonica* and *A. anguilla* transcript-to-genome alignments, the majority (~90%) of the transcripts expressed in the CS glands of *A. japonica* have 6 or less exons per transcript (Table S8).

### GO enrichment analysis and cluster classification

All the genes were analyzed according to GO functional enrichment analysis (Table 1). The top five pathways involved in molecular functions included: GTPase regulator activity, nucleoside-triphosphatase regulator activity, nucleotide binding, small GTPase regulator activity, and ATP binding. The top five biological processes included: establishment of protein localization, protein localization, protein transport, intracellular transport, and regulation of small GTPase-mediated signal transduction. The top five cellular components identified in this analysis were: intracellular organelle lumen, organelle lumen, membrane-enclosed lumen, nuclear lumen, and nucleolus.

Furthermore, the genes of the CS gland transcriptome were classified into three clusters according to their functional annotation (Table 2). Cluster I included the genes involved in the regulation of calcium metabolism such as stanniocalcin-1 (*STC-1*), calcitonin, vitamin D(3) 25-hydroxylase, calcium-sensing receptor (*CaSR*), S100 calcium-binding protein A6 (*S100A6*), and stromal interaction molecule 1 (*STIM1*). In cluster II, atrial natriuretic peptide (ANP)-converting enzyme and endothelin-converting enzyme 1 (*ECE-1*) were listed. These enzymes are involved in the proteolytic cleavage of ANP and endothelin, respectively, to produce biologically active peptides that regulate blood pressure and natriuresis. In the cluster III are those transporters involved in ion-osmoregulation such as aquaporins, chloride intracellular channel protein 5, kidney-specific Na-K-Cl symporter, and voltage-gated potassium channel subunit Kv11.1.

### Gene expression and differential gene expression

**Figure 4:**
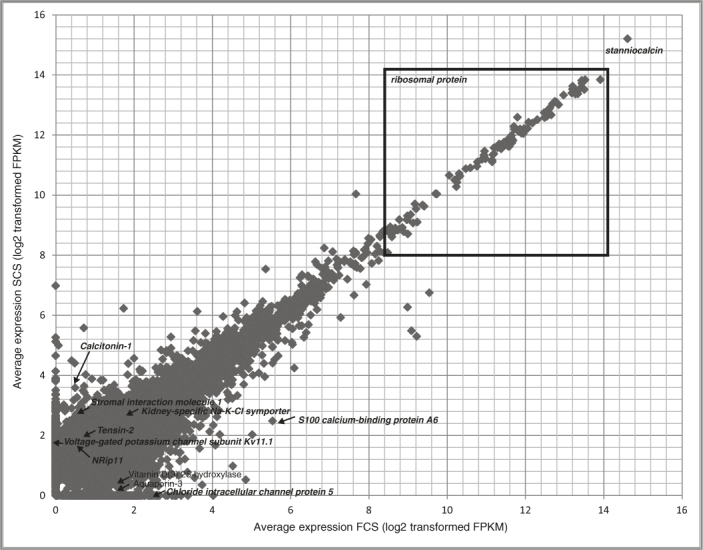
Scatter-plot of average gene expression in TMM normalized FPKM values for freshwater CS (FCS) and Seawater CS (SCS) glands.

As shown in (Fig. 4**)**, *stanniocalcin* is the highest expressed gene in CS glands^39^. Other highly expressed genes are either constituents of ribosomes or responsible for ribosome biosynthesis. The 25^th^, 50^th^, and 75^th^ quartiles of the average TMM normalized FPKM gene expression were 1.09, 1.85, and 4.34, respectively (Table S2).

By comparing the transcriptome data of the CS glands from FW and SW conditions, a total of 475 genes were identified to be differentially expressed after the transfer of fish from FW to SW (B&H corrected *p*-value <0.05 and log2 (fold change) >1). These included 357 up- and 118 down-regulated genes in the SW group compared to the FW group (Table S9). The differentially expressed genes were further analyzed using GO functional enrichment analysis (Table 3 and Table S10). The top five pathways involved in molecular functions included: diacylglycerol binding, calcium ion binding, phospholipid:diacylglycerol acyltransferase activity, cation binding, and ion binding. The top five biological process included: cell adhesion, biological adhesion, cell part morphogenesis, cell projection morphogenesis, and homophilic cell adhesion. While the top five cellular components identified in this analysis were: ubiquitin ligase complex, basolateral plasma membrane, basal plasma membrane, endoplasmic reticulum, and basal part of the cell. Ten genes involved in the functional clusters were selected and validated by qRT-PCR analysis. Primers and amplicon sizes are listed in Table S7. The results of the qRT-PCR analysis agreed with the Illumina sequencing data (Table 4).

## Discussion

The corpuscle of Stannius is a unique endocrine gland located on the ventral surface of kidneys of bony fishes. Although there is no comparable structure identified in humans, the mammalian ortholog of the CS-derived polypeptide hormone, stanniocalcin-1 (STC-1), was cloned and shown to be involved in many biological functions (i.e. ovarian physiology, inflammation, and carcinogenesis)^40^. These results demonstrated the importance of the CS-derived factor in mammals, although the development of the glands disappeared during evolution. In past studies, STC-1 is the only polypeptide identified to be responsible for the role of CS glands in Ca^2+^ homeostasis. However, physiological experiments conducted in the past decades have also demonstrated ion-osmoregulatory and pressor functions of the glands while the identities of other CS-derived active principles, surprisingly, have not been elucidated to date. In this study, we sequenced CS glands isolated from FW or SW adapted fish, assembled the transcriptome, and identified differentially expressed genes.

Our primary goal was to identify genes that explain the reported physiological importance of the CS glands in the regulation of plasma ion (Na^+^, Ca^2+^) and/or blood pressure. Nevertheless, because of the availability of extensive transcriptome and genomic resources of a closely related species, *A. anguilla*, and the draft genome of *A. japonica*, we performed a comprehensive comparison between our assembled *A. japonica* transcriptome and these resources. We found that the following peptides with hormone activity were expressed: stanniocalcin, calcitonin, activin, adrenomedullin, insulin-like growth factor, natriuretic peptide, relaxin, urotensin, and ventricular natriuretic peptide. Based on the experimental transcriptome assembly available in the EeelBase database, we estimated that more than 4085 *A. anguilla* transcripts are found in our *A. japonica* transcriptome. However, the Eeelbase specific microarray may not be suitable for analyzing transcriptome-wide expression in *A. japonica*, primarily because only hundreds of *A. japonica* transcripts could be specifically hybridized to the array’s probe. Based on the transcriptome wide predicted cDNAs available in the ZF-Genomics database, we identified 19382 putative orthologous between *A. japonica* and *A. anguilla*. We also provided a transcript-to-genome annotation of our *A. japonica* transcriptome.

Among our annotated 9254 genes, 475 genes were differentially expressed in the CS glands of SW adapted eels compared to those of FW adapted eels. GO enrichment analysis suggested that 14 differentially expressed genes mediated calcium ion binding (GO:0005509). In fish, gills and CS glands are the two major organs responsible for calcium homeostasis. A GO analysis on calcium-challenged fish gills showed that the gene category “calcium ion binding” (GO:0005509) was enriched^41^. Herein, this is the first report to identify the differentially expressed genes involved in calcium ion binding in CS glands in response to changes in environmental salinity and calcium. In addition, our data showed that a number of deregulated genes were associated with cellular protein modification (39 genes) and phosphorylation processes (10 genes). It is known that post-translational modifications are important for protein activities, stability, localization, or degradation^42^. After translation, polypeptide chains undergo modifications to produce functionally mature products. These changes are important for endocrine glands. For example, in the CS glands, they are involved in physiological signal detection and transduction via protein phosphorylation to stimulate production of STC-1 in SW adapted fish.

In addition to the general annotation, we addressed our particular research question using GO analysis to highlight the genes related to the three functional clusters: (1) Ca^2+^-metabolism, (2) blood pressure, and (3) ion-osmoregulation. The differentially expressed genes under these three functional clusters were validated using real-time PCR analysis. In fish, STC-1 is known to be a hypocalcemic hormone involved in the regulation of Ca^2+^ homeostasis^40^. However, the roles of other well-studied mammalian Ca^2+^-regulating hormones (i.e. parathyroid hormone (PTH); calcitonin (CT); and 1, 25 dihydroxyvitaminD_3_) in calcium metabolism in fish, are largely unknown. In mammals, CT is produced by parafollicular cells while in fish its presence was reported in ultimobranchial glands^43^. In this study, we firstly identified the expression of CT in CS glands. A significantly higher CT expression level was detected in the CS glands of SW adapted fish. This observation implied that CT expression responded to high salinity and/or high ambient Ca^2+^ levels. In teleost fish, the regulatory role of CT in Ca^2+^ homeostasis is not conclusive. Some studies have shown an inhibitory action of CT on GCAT in rainbow trouts^44^, suggesting a hypocalcemic function for CT. However, in another study, administration of CT caused hypercalcemia in brown trouts^45^. A recent study in zebrafish suggested that CT has a hypocalcemic function to inhibit *ECaCl* expression^46^. In mammals, CT is one of the important hypocalcemic hormones, opposing the effects of PTH, exerting inhibitory action on osteoclast, and reducing intestinal and renal Ca^2+^ (re)absorption^47^,^48^. Nevertheless, the identification of CT expression in CS glands warrants further investigation of the role of CT in plasma Ca^2+^ homeostasis in fish. Besides CT, recently the *PTH* gene family was identified in the CS glands of a cartilaginous fish, the elephant shark^49^. One of the members, *Pth1*, was found to exert PTH-like activity in mammalian UMR106.01 cells and was believed to play a fundamental role in cartilaginous fish, before evolving to regulate bone development in teleosts. Surprisingly there was no *Pth*-like transcript detected. This observation implies that PTH-producing cells may have different developmental origin than CS glands^50^. In addition to the identification of hormonal factors, our data showed an increased expression level of the stromal interaction molecule 1 (*STIM1*) in the glands of SW adapted fish. STIM1, a Ca^2+^-sensor in endoplasmic reticulum, mediates the activity of store-operated Ca^2+^ entry (SOCE) to regulate intracellular Ca^2+^ homeostasis. Upon Ca^2+^ depletion, STIM1 is translocated from the endoplasmic reticulum to the plasma membrane to activate Ca^2+^ release-activated Ca^2+ ^(CRAC) channel subunit^51^,^52^. A previous study in mammalian cells demonstrated that the function of the STC-1 paralog, STC-2, was to interact with STIM1 to negatively modulate SOCE^53^. Thereby, STIM1 may play a role in mediating the signal of extracellular Ca^2+^ to modulate STC-1 synthesis in CS glands.

In addition to the Ca^2+^-regulatory function, early studies of CS gland physiology denoted the presence of pressor substances. In STX fishes, a decrease of dorsal aortic blood pressure was reported^18^. An injection of CS extracts increased blood pressure of fish^54^. However, the pressor substances in the glands that increased systemic blood pressure remain unknown. Through this transcriptomic analysis, we identified the expression of atrial natriuretic peptide (ANP)-converting enzyme and endothelin-converting enzyme 1 (*ECE-1*). Although no significant difference in their mRNA expression levels in CS glands was measured between the FW and SW adapted fish, the two enzymes are known to indirectly regulate blood pressure. The ANP-converting enzyme is an endopeptidase that cleavages atrial natriuretic peptide hormone into an active form to promote natriuresis and vasodilation^55^. ECE-1 is involved in proteolytic activation of endothelins, which have strong vasoconstrictive effects^56^. The identification of these important enzymes suggests the involvement of the glands in the regulation of blood pressure in fish and provides an explanation to the pressor effects of the gland extracts.

The functional cluster named “ion-osmoregulation” was formed by identifying the changes in the expression levels of the membrane transporters which were interpreted as the modulation of membrane sensors to integrate extracellular signals to regulate CS gland functions. In particular, the expression level of *AQP-3* was significantly reduced in the CS glands of SW fish. The studies of the functions of water-specific, membrane-channel AQP proteins in mammals and fish suggested that AQPs have unique permeability characteristics, are widely distributed across tissues, and play important roles in the regulation of water homeostasis^57^,^58^. AQPs are functionally classified as osmotic-stress effectors. A long-term osmotic-stress in oysters induced a reduction of AQPs activities in response to osmotic challenges^59^. The reduced *AQP-3* expression in the CS glands of SW fish might serve to similar functions.

In summary, our work represents the first report using next generation sequencing to identify gene targets that could explain the reported physiological importance of the CS glands. Three functional clusters were defined and differential gene expression was observed in the CS glands of fish adapted to FW and SW conditions. Taken together, our data support the notion that CS glands are important in the regulation of ion homeostasis and blood pressure. It warrants further investigation to decipher the underlying mechanisms that characterize the additional functions of this unique endocrine gland.

## Authors contributions

KPL participated in next generation sequencing experiments and drafted the manuscript, JWL and TFC carried out the transcriptome data analysis and drafted the manuscript, JG carried out real time PCR analysis and drafted the manuscript, WKFT participated in samples collection and samples preparation for transcriptome sequencing, CKCW participated in the design of the study and drafted themanuscript. JWL and JG contributed equally in this work. All authors read and approved the final manuscript.

## Supplementary Material

Supplementary Information Supplementary Information

Supplementary Information Supplementary Information

Supplementary Information Supplementary Information

Supplementary Information Supplementary Information

Supplementary Information Supplementary Information

Supplementary Information Supplementary Information

Supplementary Information Supplementary Information

Supplementary Information Supplementary Information

Supplementary Information Supplementary Information

Supplementary Information Supplementary Information

Supplementary Information Supplementary Information

## Figures and Tables

**Table 1:** GO classification of genes from CS gland of *Anguilla japonica*.

Term	gene	*p*-Value
**Molecular function**		
GTPase regulator activity (GO:0030695)	168	6.20E-22
nucleoside-triphosphatase regulator activity (GO:0060589)	170	1.20E-21
nucleotide binding (GO:0000166)	616	1.50E-17
small GTPase regulator activity (GO:0005083)	118	5.10E-17
ATP binding (GO:0005524)	424	8.40E-15
**Biological process**		
establishment of protein localization (GO:0045184)	258	3.40E-18
protein localization (GO:0045184)	287	4.10E-18
protein transport (GO:0045184)	254	1.60E-17
intracellular transport (GO:0046907)	217	2.50E-14
regulation of small GTPase mediated signal transduction (GO:0051056)	100	5.40E-12
**Cell component**		
intracellular organelle lumen (GO:0070013)	503	9.60E-22
organelle lumen (GO:0043233)	506	3.30E-20
membrane-enclosed lumen (GO:0031974)	514	3.60E-20
nuclear lumen (GO:0031981)	418	1.60E-19
Nucleolus (GO:0005730)	216	3.10E-13

**Table 2:** Classification of genes from CS glands of *Anguilla japonica* based on three reported physiological functions.

Gene	Transcript ID
**Cluster I Ca^2+^ metabolism** (Total 492 genes)	
Stanniocalcin-1	comp31909_c2
Calcitonin-1	comp18601_c0
Vitamin D(3) 25-hydroxylase	comp7276_c0
Calcium-sensing receptor	comp610_c0
S100 calcium-binding protein A6	comp22265_c0
Stromal interaction molecule 1	comp32767_c0
**Cluster II Blood pressure** (Total 26 gene)	
Endothelin-converting enzyme 1	comp30404_c0
Atrial natriuretic peptide-converting enzyme	comp2660_c0
**Cluster III Ion-osmoregulation** (Total 101 gene)	
Aquaporin-1	comp21702_c0
Aquaporin-3	comp12261_c0
Chloride intracellular channel protein 5	comp21102_c0

**Table 3:** GO classification of the differentially expressed genes from CS glands of *Anguilla japonica*.

Term	Count Total (up/down)	p-Value
**Molecular function**		
Diacylglycerol binding (GO:0019992)	5(3/2)	2.21E-03
Calcium ion binding (GO:0005509)	14(11/3)	3.39E-02
Phospholipid:diacylglycerol acyltransferase activity (GO:0046027)	2(2/0)	3.98E-02
Cation binding (GO:0043169)	43(34/9)	5.49E-02
Ion binding (GO:0043167)	43(34/9)	6.79E-02
**Biological process**		
cell adhesion (GO:0007155)	16(14/2)	4.79E-04
Biological adhesion (GO:0022610)	16(14/2)	4.86E-04
Cell part morphogenesis (GO:0032990)	9(8/1)	1.08E-03
Cell projection morphogenesis (GO:0048858)	8(7/1)	3.65E-03
Homophilic cell adhesion (GO:0007156)	6(5/1)	4.13E-03
**Cell component**		
Ubiquitin ligase complex (GO:0000151)	7(6/1)	1.57E-04
Basolateral plasma membrane (GO:0016323)	7(7/0)	1.00E-02
Basal plasma membrane (GO:0009925)	3(3/0)	2.27E-02
Endoplasmic reticulum (GO:0005783)	16(14/2)	2.50E-02

**Table 4:** Relative mRNA expression of 10 selected genes for comparison of the SW versus FW groups, in respect to RNA-Seq and real-time PCR.

Gene	Transcript ID	Real-time PCR (log2 fold change)	Illumina RNA-seq (log2 fold change)			Quartile of RNA-Seq gene expression ˆ
**Ca^2+^ metabolism**						
Calcitonin-1	comp18601_c0	3.79±0.19 *		3.58		75^th^
Vitamin D(3) 25-hydroxylase	comp7276_c0	−5.73±1.23 *		−3.37		50^th^
S100 calcium-binding protein A6	comp22265_c0	−1.58±0.44 *		−3.66		75^th^
Tensin-2	comp12417_c0	1.72±0.35 *		1.99		50^th^
Stromal interaction molecule 1	comp32767_c0	1.60±1.04 *		1.78		25^th^
**Ion-osmoregulation**						
Chloride intracellular channel protein 5	comp21102_c0	−5.54±1.72 *		−6.70		75^th^
Kidney-specific Na-K-Cl symporter	comp29031_c0	6.63±0.40 *		2.18		75^th^
Voltage-gated potassium channel subunit Kv11.1	comp155490_c0	3.88±0.47 *		5.51		50^th^
Aquaporin-3	comp12261_c0	−7.98±3.06 *		−3.91		50^th^
NRip11	comp22623_c0	1.53±0.64 *		2.08		50^th^

*Asterisk indicates statistical significance of differential gene expression with p-value <0.05.

ˆ Quartile of the gene expression of the group with higher expression. For example, Calcitonin-1 is induced by sea water treatment, and the quartile of average expression of SW was reported.
