# Supplementary material for: Transcriptomic responses of corpuscle of Stannius gland of Japanese eels (Anguilla japonica) to Changes in Water Salinity
Source: Sci Rep. 2015 Apr 24;5:9836. doi: 10.1038/srep09836 (PMC5386212; doi:10.1038/srep09836)
Supplement: Supplementary Information [file srep09836-s11.doc]

**Title of manuscript: Transcriptomic responses of corpuscle of Stannius gland of Japanese eels (*Anguilla japonica*) to Changes in Water Salinity**

Author list:

Jie Gu^1§^,, Jing-Woei Li^2§^, William Ka-Fai Tse^1^, Ting-Fung Chan^2^, Keng-Po Lai^3*^, and Chris Kong-Chu Wong^1*^

^1^ Department of Biology, Hong Kong Baptist University, Hong Kong SAR, China;

^2^School of Life Sciences, Hong Kong Bioinformatics Centre, The Chinese University of Hong Kong, Hong Kong SAR, China;

^3^School of Biological Sciences, Kadoorie Biological Sciences Building, The University of Hong Kong, Pokfulam Road, Hong Kong SAR, China;

^§^ These authors contributed equally to this work.

***** *Corresponding author*

Email address:

Jie Gu: 10467548@life.hkbu.edu.hk

Jing-Woei Li: [marcoli@cuhk.edu.hk](mailto:marcoli@cuhk.edu.hk)

William Ka-Fai Tse: kftse@hkbu.edu.hk

Ting-Fung Chan: tf.chan@cuhk.edu.hk

Chris Kong-Chu Wong: ckcwong@hkbu.edu.hk

Keng-Po Lai: balllai@hku.hk

**Supplementary Table Legends**

**Table S1.** Primer sequences and amplicon sizes of genes used for realtime RT-PCR. (XLSX)

**Table S2.** Gene annotation and expression level of the assembled **transcripts from** CS glands of *Anguilla japonica*. (XLSX)

**Table S3.** Recovery of Anguilla proteins with hormone activity deposited in UniProt. (XLSX)

**Table S4.** Recovery of Anguilla mRNA sequences deposited in NCBI. (XLSX)

**Table S5.** Recovery of Anguilla proteins sequences deposited in the automatically annotated TrEMBL of UniProt. (XLSX)

**Table S6.** Analysis of japonica transcriptome assembly with respective to Eeelbase transcripts and Eeelbase specific microarray. (XLSX)

**Table S7.** Putative homology between *japonica* and *anguilla* identified using reciprocal BLAST. (XLSX)

**Table S8.** Transcript-to-genome annotation of the *japonica* transcriptome generated in this study. (XLSX)

**Table S9.** Differentially expressed genes in CS glands of *Anguilla japonica* of the SW group versus FW group. (XLSX)

**Table S10.** GO classification of the differentially expressed genes in CS glands of *Anguilla japonica* of SW group compared with FW group. (XLSX)
